# Physical activity and quality of life in severely obese individuals seeking bariatric surgery or lifestyle intervention

**DOI:** 10.1186/1477-7525-10-86

**Published:** 2012-07-28

**Authors:** Dale S Bond, Jessica L Unick, John M Jakicic, Sivamainthan Vithiananthan, Jennifer Trautvetter, Kevin CO’Leary, Rena R Wing

**Affiliations:** 1 Department of Psychiatry and Human Behavior, The Miriam Hospital/Weight Control and Diabetes Research Center, Warren Alpert Medical School of Brown University, 196 Richmond Street, Providence, RI, 02903, USA; 2 Department of Health and Physical Activity, Physical Activity and Weight Management Research Center, University of Pittsburgh, Pittsburgh, PA, USA; 3 Department of Surgery, The Miriam Hospital, Warren Alpert Medical School of Brown University, Providence, RI, USA

**Keywords:** Bariatric surgery, Lifestyle intervention, Physical activity, Sedentary, Health-related quality of life, Sensewear armband

## Abstract

**Background:**

Given that bariatric surgery (BS) and lifestyle intervention (LI) represent two vastly different approaches to treating severe obesity, there is growing interest in whether individuals who seek BS versus LI also differ on weight-related behaviors. In the present study, we compared BS- and LI-seekers on physical activity (PA) and sedentary behaviors (SB), and examined between-group differences in health-related quality of life (HRQoL), while controlling for PA.

**Findings:**

A sample of 34 LI-seekers were matched with 34 BS-seekers on gender, age, BMI, and PA monitor-daily wear time (age: 42.1±10.0 years; BMI: 45.6±6.5 kg/m^2^). PA and SB were assessed over a 7-day period via the SenseWear Armband (SWA). HRQoL was measured using the SF-36, with scores standardized to a population normal distribution (M=50, SD=10). Participants wore the SWA for 13.7±1.6 h/day. BS-seekers did not differ from LI-seekers on average min/d over the wear period spent in SB (641±117.1 vs. 638.4±133.4, *p*=0.62) or light (136.4±76.1 vs. 145.5±72.5, *p*=0.59) and moderate-to-vigorous (>1-min bouts=36.4±26.2 vs. 40.2±31.3, *p*=0.59; ≥10-min bouts=5.7±8.3 vs. 10.2±17.0, *p*=0.17) PA. BS-seekers reported significantly lower SF-36 physical functioning (42.4±10.9 vs. 49.0±6.8, *p*=0.004) and physical component summary (43.9±10.1 vs. 48.9±7.0) scores versus LI-seekers. BS-seeker group status was related to lower physical functioning (β=0.30, *p*=0.009), independent of gender, age, BMI, and daily PA.

**Conclusions:**

Findings suggest that seeking BS versus LI is not related to patterns of PA or SB, and that lower subjective physical functioning is not associated with lower overall PA levels in BS-seekers.

## Introduction

Two options for treating severe obesity are bariatric surgery and lifestyle intervention. To achieve weight loss, bariatric surgery procedures force modification of eating behavior and alter appetite regulation via surgical restriction of food intake, inducing nutrient malabsorption, and producing favorable changes in gut peptides/hormones [1]. Conversely, the goal of lifestyle intervention, a more conservative approach, is to promote voluntary modification of eating and physical activity behaviors to lose weight [2].

Given that bariatric surgery and lifestyle intervention are substantially different approaches to weight loss, there is a question of whether individuals who seek bariatric surgery versus lifestyle interventions may also differ in important ways. Research has shown that bariatric surgery-seekers, compared to lifestyle intervention-seekers, are heavier, more often diagnosed with comorbidities, and report poorer health-related quality of life (HRQoL) impairments [3-5]. However, it is less clear whether these groups also differ on weight-related behaviors. Studies comparing eating behaviors of bariatric surgery- and lifestyle intervention-seekers have produced mixed findings [3,5]. To our knowledge, no study has compared physical activity and sedentary behaviors in these groups.

Our previous research using objective measures shows that bariatric surgery-seekers have low physical activity levels and spend 80% of their time in sedentary behaviors [6,7]. However, it is not known whether equally overweight individuals seeking to lose weight via lifestyle intervention have similar levels of physical activity and sedentary behaviors. Thus, the primary aim of this study was to compare objectively-measured daily time spent in physical activity and sedentary behaviors between bariatric surgery- and lifestyle intervention-seekers. Additionally, while previous research has found that lower physical activity is associated with worse HRQoL in bariatric surgery-seekers [8,9], this has not been shown in lifestyle intervention-seekers. Thus, a secondary aim was to examine HRQOL differences between these groups, while controlling for physical activity, body mass index (BMI) and demographics.

## Methods

### Participants and procedures

Participants were 21–65 years of age, with a BMI ≥ 35 kg/m^2^ and no medical contraindications for physical activity, who were seeking to lose weight via bariatric surgery or lifestyle intervention. Participants seeking bariatric surgery in the current study were initially recruited from local surgery clinics and patient support groups to participate in a wider study investigating behavioral changes in this population. Lifestyle intervention-seekers were recruited from the community via internet and newspaper advertisements. Prior to treatment, both groups completed questionnaires and were given an activity monitor to wear for 7 consecutive days. Study procedures were approved by The Miriam Hospital institutional review board (Providence, RI, USA).

### Measures

#### Objective physical activity

The SenseWear Armband (SWA; BodyMedia, Pittsburgh, PA) simultaneously integrates motion data from a biaxial accelerometer and physiological metrics from multiple sensors to provide minute-by-minute estimates of energy expenditure at different intensity levels. The SWA has been shown to produce valid energy expenditure estimates when evaluated against indirect calorimetry and doubly labeled water [10,11]. For data to be considered valid, participants had to wear the SWA for ≥8 h/d on 4 days. The intensity of activity for each minute of wear time was calculated and expressed using metabolic equivalents (METs) to determine average daily time (min/d) spent in sedentary (<1.5 METs) activities, and light (1.5-2.9 METs), moderate-to-vigorous (MVPA: ≥3 METs; ≥1 and ≥10-min bouts), and total (≥1.5 METs) PA.

#### HRQoL

The Medical Outcomes Study Short Form-36 (SF-36) is a widely used and psychometrically sound HRQoL measure. Scores from 8 domains relating to physical (physical function, role-physical, bodily pain, general health) and mental (vitality, social functioning, role-emotional, mental health) health are individually weighted into physical (PCS) and mental (MCS) component summary scales. Scores are transformed to a 0–100 scale and standardized to a population normal distribution (M = 50, SD = 10), with higher scores representing better HRQoL [12,13].

### Statistical Analysis

Analyses were performed using SPSS for Windows (Version 18.0, Chicago, IL). A sample of 34 lifestyle intervention-seekers was matched with 34 bariatric surgery-seekers, selected from a wider study database (n = 130), on gender, age, BMI, and daily SWA wear time. Independent *t*-tests examined group differences in continuous variables. Chi-square analyses compared proportions across categories. Linear regression examined the association of group status (bariatric surgery- vs. lifestyle intervention-seekers) with HRQoL, adjusting for total physical activity min/d, BMI, age, and sex.

## Results

### Participants

As shown in Table 1, bariatric surgery-seekers and lifestyle intervention-seekers did not differ significantly on demographics or weight characteristics. Overall, participants on average were 42.1±10.0 years of age, mostly female (76.5%) and non-Hispanic White (75%), with BMI=45.6±6.5 kg/m^2^ and weight=128.8 ±23.8 kg.

**Table 1:** Characteristics of severely obese participants seeking bariatric surgery or lifestyle intervention

	**Bariatric Surgery (N = 34)**	**Lifestyle Intervention (N = 34)**	***P***
Age (yrs)	43.3 ± 9.4	40.9 ± 10.5	0.33
% Female	76.5	76.5	1.00
% Race			0.25
White	79.4	70.6	
African-American	5.9	14.7	
American Indian/Alaskan Native	2.9	0.0	
Native Hawaiian/Other Pacific Islander	2.9	0.0	
Other	8.8	5.9	
Don’t know	0.0	8.8	
% Ethnicity			1.00
Non-Hispanic	91.2	91.2	
Hispanic	8.8	8.8	
BMI (kg/m^2^)	46.2 ± 7.8	45.0 ± 4.9	0.49
Weight (kg)	129.9 ± 27.6	127.7 ± 19.6	0.71

Note. *P* values are based on independent samples t-tests for continuous variables and χ^2^ tests for categorical variables. BMI = body mass index.

### Differences in PA and SB

Participants wore the SWA for 13.7±1.6h/day over a 7-day period. Table 2 shows that bariatric surgery- and lifestyle intervention-seekers did not differ in average daily min/d spent performing sedentary behaviors and physical activity of different intensities (*p*’s>0.50).

**Table 2:** Comparison of time spent performing activities of varying intensity and reported health-related quality of life between severely obese participants seeking bariatric surgery or lifestyle intervention

	**Bariatric Surgery (N = 34)**	**Lifestyle Intervention (N = 34)**	***P***
**Activity intensity (METs)**			
Sedentary (min/d)	641.0 ± 117.7	638.4 ± 133.4	0.62
Light (min/d)	136.4 ± 76.1	145.5 ± 72.5	0.59
Moderate-to-vigorous (min/d)	36.4 ± 26.2	40.2 ± 31.3	0.59
Total physical activity (min/d)	172.8 ± 94.3	185.7 ± 94.1	0.58
**Health-Related Quality of Life (SF-36)**			
Physical function	42.4 ± 10.9	49.0 ± 6.8	**0.004**
Role-physical	46.7 ± 11.1	49.0 ± 10.7	0.39
Bodily pain	50.6 ± 7.6	52.8 ± 7.1	0.22
General health	41.8 ± 9.6	42.3 ± 8.6	0.83
Physical component summary	43.9 ± 10.1	48.9 ± 7.0	**0.02**
Vitality	46.2 ± 10.0	47.0 ± 10.1	0.12
Social functioning	46.6 ± 8.6	47.6 ± 10.3	0.73
Role-emotional	49.1 ± 10.1	48.2 ± 12.1	0.52
Mental health	49.5 ± 9.0	48.1 ± 10.0	0.54
Mental component summary	49.1 ± 9.3	47.2 ± 11.3	0.44

Note. *P* values are based on independent *t*-tests. Values are presented as mean ± standard deviation. METs = metabolic equivalents. Activity intensity (METs) thresholds = sedentary (<1.5 METs), light (1.5-2.9 METs), moderate-to-vigorous (≥3 METS), and total physical activity (≥1.5 METs). SF-36 scores are standardized to a population normal distribution with a mean of 50 and standard deviation of 10 to provide context for reported degree of health-related quality of life impairment [12].

Additionally, overall, the groups did not differ in average daily moderate-to-vigorous physical activity minutes accumulated in ≥10-min bouts across the 7-day wear period (bariatric surgery-seeker: 5.7±8.3 vs. lifestyle intervention-seeker: 10.2±7.1, *p*=0.17). However, a significantly smaller proportion of bariatric surgery-seekers performed at least one 10-min bout versus lifestyle intervention-seekers (47.1%/n=16 vs. 82.4%/n=28, *p*=0.005). Among these participants, the groups did not differ in the number (bariatric surgery-seeker: 5.8±3.4 vs. lifestyle intervention-seeker: 6.8±9.4, *p*=0.68) or average duration (bariatric surgery-seeker: 14.4±4.9 vs. lifestyle intervention-seeker: 13.4±3.9, *p*=0.46) of bouts performed.

### Differences in HRQoL

Bariatric surgery-seekers reported lower scores on the physical function domain and the PCS, compared to lifestyle intervention-seekers (*p*’s<0.05) (Table 2). In regression analyses, bariatric surgery-seeker group status was associated with lower physical function scores (β=0.30, *p*=0.009), independent of gender, age, BMI, and total PA min/d [Model R^2^=0.20, *F* (5, 67)=3.1, *p*=0.02]. The SF-36 PCS model was not significant.

## Discussion

Given that bariatric surgery is the most aggressive approach for treating severe obesity, there is interest in whether individuals who seek bariatric surgery engage in less favorable weight-control behaviors than those who opt for a less aggressive lifestyle intervention approach. However, previous research in this area has been limited to eating behaviors [3,5].

The current study compared daily time spent in physical activity and sedentary behaviors among individuals seeking bariatric surgery versus lifestyle intervention. We found that these groups exhibited a remarkably similar profile characterized by high levels of sedentary behavior and infrequent engagement in structured or bout-related moderate-to-vigorous physical activity. Overall, bariatric surgery- and lifestyle intervention-seekers engaged in similar low levels of bout-related moderate-to-vigorous physical activity; however, a greater proportion of lifestyle intervention-seekers actually performed this type of activity. Future studies are needed to investigate possible barriers to bout-related moderate-to-vigorous physical activity in bariatric surgery-seekers (e.g., low motivation, confidence, fear of injury, etc.) that may account for this difference [14].

We also compared bariatric surgery- and lifestyle intervention-seekers on HRQoL. Similar to previous studies [4,5], bariatric surgery-seekers reported lower physical HRQoL than lifestyle intervention-seekers. Interestingly, however, this difference occurred despite the groups having similar body weight and physical activity levels. While reasons for this difference are not entirely clear, one possibility may be that lower perceived physical functioning is a primary reason why severely obese individuals opt for bariatric surgery, a more aggressive treatment modality, over lifestyle intervention, a more conservative treatment modality. It is also possible that this finding is due in part to differences between the groups on actual or perceived health indicators (e.g., duration of severe obesity, severity of weight-related comorbidities) that were not directly measured.

Importantly, the above finding also suggests that lower subjective physical functioning in bariatric surgery-seekers is not a barrier to PA. This discordance between subjective and objective physical functioning in bariatric surgery-seekers was also shown in another recent study, where 41 % of bariatric surgery-seekers reported having walking limitations yet did not demonstrate limitations during an objective walking test [9].

Study strengths include use of an objective physical activity measure and matching of lifestyle intervention and bariatric surgery participants on potential confounding variables. This study also has certain limitations including relatively small sample size, non-random assignment of participants, and exclusion of participants with medical contraindications for physical activity, that may limit generalizability to all severely obese individuals.

In summary, to our knowledge, this is the first study to compare severely obese individuals seeking bariatric surgery or lifestyle intervention on patterns of physical activity and sedentary behaviors. We found that these groups engaged in similar levels of physical activity and sedentary behaviors, and that lower subjective physical functioning among bariatric surgery-seekers was not associated with lower overall physical activity levels. The results suggest that similar prescriptions can be used to increase physical activity and decrease sedentary behavior in severely obese individuals seeking bariatric surgery or lifestyle intervention. Further research is needed to determine whether interventions can produce similar physical activity and sedentary behavior changes in these groups.

## Competing Interests

The authors declare that they have no competing interests.

## Author Contributions

DSB, JLU, JMJ and RRW conceived of the design of the study and contributed to analysis and interpretation of the data. SV, JT, and KO contributed to data acquisition and analysis of data. DB drafted the manuscript and all authors assisted in revising it critically for important intellectual content. All authors gave final approval of the version to be published.
